# A semi-centralized blockchain system with multi-chain for auditing communications of Wide Area Protection System

**DOI:** 10.1371/journal.pone.0245560

**Published:** 2021-01-22

**Authors:** Yong Wang, June Li, Yunsong Yan, Xiong Chen, Fajiang Yu, Siyu Zhao, Tongwei Yu, Ke Feng

**Affiliations:** 1 Key Laboratory of Aerospace Information Security and Trusted Computing, Ministry of Education, School of Cyber Science and Engineering, Wuhan University, Wuhan, China; 2 State Grid Electric Power Research Institute, Nanjing, China; 3 Electric Power Research Institute of State Grid Liaoning Electric Power Co., Ltd., Shenyang, China; Northeastern University at Qinhuangdao, CHINA

## Abstract

Wide Area Protection System (WAPS) undertakes the important task of maintaining system reliability and stability when the power system is subject to abnormal or predetermined unstable conditions. The existing WAPS adopts a centralized mechanism to record and audit communication messages, which faces the risk of excessive authority and tampering with communication records and audit logs, thus making it impossible to achieve true transparency and fairness. Due to the involvement of multiple parties and equipment maintained by different manufacturers in the communication of WAPS, there are difficulties in tracing the cause of the accident and determining the at-fault party following misoperations and miss trips. To address this issue, we propose a semi-centralized blockchain system with multi-chain for auditing communications of WAPS. We first propose a semi-centralized system architecture according to the system architecture and management requirements of WAPS. Then, we utilize the blockchain network as a self-recording channel to achieve tamper-proof and non-repudiation verification interaction. We also design a multi-chain structure and classification node mechanism to meet the communication auditing requirements of multiple WAPS. We have designed a new block structure that conforms to the communication protocol of WAPS. To reduce the storage burden caused by the ever-expanding blockchain ledger, we propose a deletable blockchain scheme while maintaining the integrity and security of blockchain. Analysis and experiments show that the proposed blockchain system can support the secure, transparent, tamper-proof and traceable communication recording and auditing of WAPS along with high performance.

## 1. Introduction

With the scale and operation complexity of power grid ever-increasing, the power system is vulnerable and subject to system wide disturbance, which may cause the power system to collapse in un-planned emergency conditions [1]. To solve this system wide operating problem, Wide Area Protection System (WAPS) [2], also known as System Protection Scheme (SPS) [3], Remedial Action Scheme (RAS) [4] and Security and Stability Control System (SSCS) [5] in China, is developed to address this issue. WAPS utilizes a set of automatic and fast control actions, protection relays and telecommunications network to ensure the reliability, security and stability of the power system following critical outages on a transmission network [6]. The communication messages of WAPS include measurement data and control commands, such as sampling values of voltage, fault information, low frequency load shedding and tap changer control. The normal communication of WAPS requires a high degree of coordination between multiple parties and equipment maintained by different manufacturers. When a misoperation or miss trip occurs, there are difficulties in tracing the cause of the accident and determining the at-fault party. Hence, it is very important for WAPS to record and audit these communication messages.

### 1.1 Motivation

The existing WAPS adopts a centralized mechanism to record and audit communication messages, which is vulnerable to many security threats. (i) The existing audit systems suffer from excessive authority of privileged accounts. The internal privileged accounts can tamper with the audit data without leaving any traces, and auditors are unable to recover the modified audit data, thus failing to get true and effective audit evidence [7]. (ii) The external attackers can maliciously tamper with communication records and audit logs without being detected [8]. There are two well-known cyber attacks that seriously threaten the communication of WAPS, namely the physical access attack [9] that exploits root privileges and the remote vulnerability attack [10] that exploits known vulnerabilities in database system. To this end, there is an urgent practical need for a secure, transparent, tamper-proof and traceable communication recording and auditing system of WAPS.

The emergence and rapid advancement of blockchain technology provides a new solution for communication recording and auditing of WAPS. The technical features of blockchain, such as decentralization, transparency, multi-party consensus, tamper-proof, traceability and irrevocability [11], can naturally make up for the defects of existing communication recording and auditing system. (i) The distributed system architecture of blockchain eliminates the need for centralized third parties and enhances the efficiency, security and reliability of audit procedures [12]. (ii) Blockchain network can be used as a self-recording channel to achieve tamper-proof and non-repudiation verification interaction. (iii) The chained data structure of blockchain enables the full lifecycle traceability of audit data and ensures the authenticity, accuracy and integrity of audit data. (iv) The timestamp mechanism of blockchain can improve the data collection and screening process of audit system and improve the efficiency of obtaining audit evidence. (v) The multi-party consensus mechanism of blockchain can ensure that blocks containing the latest communication messages are accurately added to the blockchain, the blockchain ledger stored by all the nodes is consistent without forking [13]. (vi) The maturity of blockchain in terms of speed, energy consumption, interoperability and cost-effectiveness is rapidly evolving. WAPS rarely sends down control commands in practice, so the frequency of recording and auditing control commands sent by WAPS is also very low. The performance of existing blockchain system is sufficient to meet the requirement of such low-frequency transactions.

### 1.2 Related work

Scholars have proposed a variety of methods to apply blockchain to the audit system. Shigeya Suzuki et al. [14] proposed a scheme to set blockchain as an auditable communication channel among multi-party. By putting a message marked with the receiver’s identity onto a blockchain based communication channel, anyone who has access to the blockchain can verify this message. However, the transaction can be modified by malleability attack. Jing Chen et al. [15] proposed CertChain, a blockchain-based certificate audit scheme for secure SSL/TLS connections. To ensure the traceability and efficient query response, it proposed a new data structure called CertOper to record certificate operations. Shi-Cho Cha et al. [16] proposed a security auditing system that compliant with ISO/IEC 15408–2 by taking blockchain network as the underlying communication architecture. The proposed system not only inherits the advantages of blockchain such as decentralization, transparency, immutability, and traceability, but also meets the critical audit requirements identified by ISO/IEC 15408–2. Nesrine Kaaniche et al. [17] proposed BDUA, a new data usage auditing system based on blockchain that ensures the control and privacy protection of distributed data exchange, thus authorized auditing entities can rely on registered blockchain transactions for accurate auditing. Haiyang Yu et al. [18] proposed a big data auditing scheme for the cloud based on blockchain. This study designed a new blockchain instantiation called Data Auditing Blockchain (DAB) to improve the reliability and stability of the auditing scheme. Yang Xu et al. [19] presented a blockchain-based arbitrable data auditing scheme for network storage. This study proposed a data audit protocol with credible arbitration to prevent malicious verifiers from cheating undetectably. Guofang Dong et al. [20] presented a secure IoT data integrity auditing scheme based on Hyperledger Fabric to solve the centralized problem of trusted Third Party Auditor (TPA). This study used blockchain as a three-way communication tool and adopted the smart contract to publish task. Ying Miao et al. [21] proposed DBPA, a blockchain-based public auditing scheme that can provide users with security against challenge messages guessing attacks and privacy protection during the audit process. Ashar Ahmad et al. [22] proposed BlockAudit, an end-to-end solution that combines audit log with blockchain system. BlockAudit converted the audit logs into a blockchain-compatible data structure. It then created timestamped transactions from data within the audit logs and aggregates them in a block. Besides, Ashar Ahmad et al. [23] also proposed BlockTrail, an efficient and scalable blockchain scheme for audit trail applications that leverages the multichain blockchain model to provide tamper-proof audit trails. Danilo Francati et al. [24] proposed Audita, a flexible blockchain-based storage system that assures data protection and solves challenges such as privacy and scalability. Audita worked through an enhanced blockchain network of participants, including storage nodes and block creators.

However, the current blockchains are primarily designed for transaction applications. Their architectures, algorithms and communication protocols are not applicable to WAPS with limited computation and storage resources. There is no known scheme that applies existing blockchain to the communication recording and auditing of WAPS. Therefore, it is essential to design a novel blockchain system for auditing communications of WAPS.

### 1.3 Contributions

The original contributions of this paper include:

1. We propose a semi-centralized architecture for auditing communications of WAPS according to the system architecture and management requirements of WAPS.
2. We design a multi-chain structure and classification node mechanism that meets the communication auditing requirements of multiple WAPS. The adoption of multi-chain structure and classification node mechanism can effectively reduce computation resource consumption and save storage resources of system nodes.
3. We design a new block structure that conforms to the communication protocol of WAPS. We redesign the block header and block body of the blockchain structure, and transform the communication records and audit logs into a blockchain-compatible data structure for storage.
4. We design a deletable blockchain to reduce the storage burden of WAPS while maintaining the integrity and security of blockchain. We extend the block structure by adding a *MerkleRootCopy* field in the block header to store a copy of the *MerkleRoot* of the original block data. We have validated the security of proposed deletable blockchain scheme through a detailed security analysis.

The remainder of this paper is organized as follows. A brief introduction of WAPS and blockchain is given in Section 2. The semi-centralized system architecture, the multi-chain structure and new block structure of the proposed blockchain system are introduced in Section 3. Then, a novel deletable blockchain for communication recording and auditing of WAPS is proposed in Section 4. The security analysis of the proposed blockchain system is shown in Section 5. A series of performance experiments are carried out to demonstrate the feasibility of the innovative proposal in Section 6. Finally, Section 7 concludes the work and highlights future research directions.

## 2. Preliminaries

### 2.1 Wide Area Protection System

WAPS is positioned as a system protection and control method between conventional protection and SCADA/EMS [25]. A comprehensive WAPS consists of many control features, such as Under-Frequency Load Shedding (UFLS), generation rejection, tap changer control, under-voltage load shedding, load rejection and Flexible AC Transmission System (FACTS) [26], etc. Compared with the traditional stability control strategy, WAPS involves a wider geographical range and requires more complex calculations in the process of obtaining information, forming control strategies and executing control measures.

**Fig 1:**
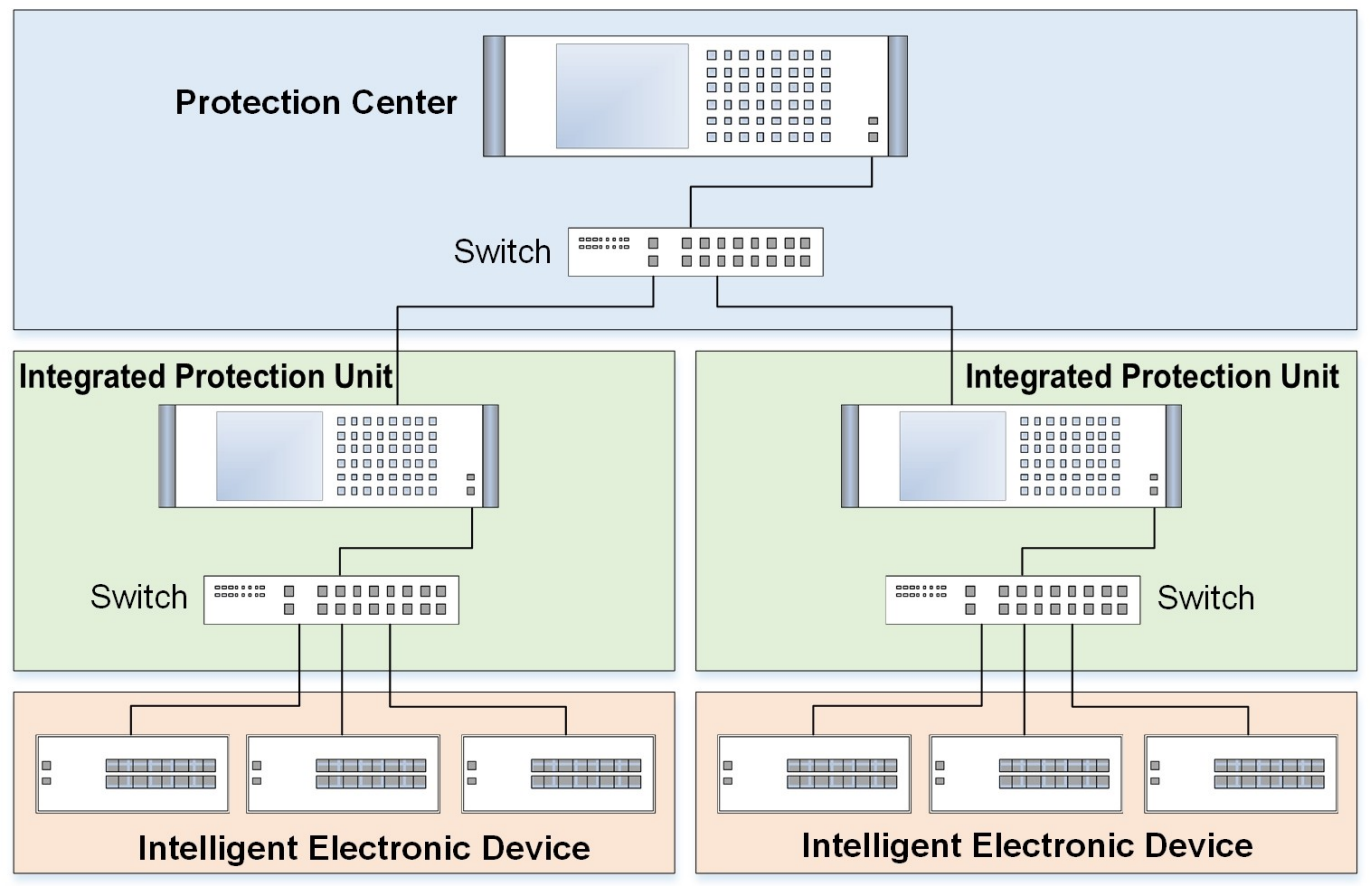
Communication architecture of WAPS.

As shown in Fig 1, the communication architecture of WAPS consists of Protection Center (PC), Integrated Protection Unit (IPU) and Intelligent Electronic Device (IED) [27]. The PC is responsible for managing the overall network and sending out control commands to IPUs and IEDs via communication channel. The IPU is a small autonomous protection system that enables self-control, protection and management. The IPU is responsible for coordinating and monitoring the IEDs and protection relays. The IEDs are used to achieve the function of switch control and communication, conventional primary protection, electrical parameter computation, digital and analog signal acquisition.

### 2.2 Blockchain

**Fig 2:**
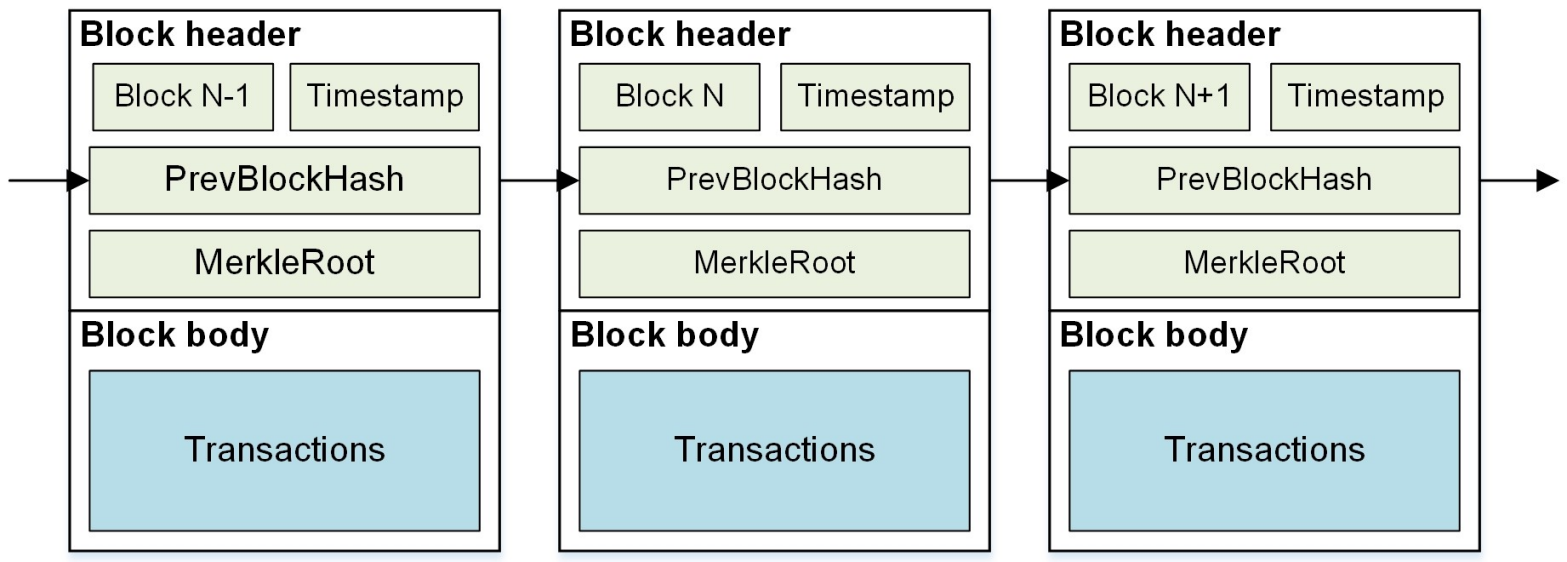
Blockchain data structure consisting of three blocks.

The idea of blockchain was proposed by Satoshi Nakamoto for the bitcoin system in 2008 [28], and the concept of blockchain first appeared in 2015. Blockchain has many advantages such as decentralization, transparency, tamper-proof, traceability, irrevocability and verifiability [29]. Blockchain is a virtual chain of chronologically ordered blocks. Each valid node keeps a copy of the blockchain ledger and uses a consensus mechanism to make all nodes agree on the content of the transaction data, thus avoiding the problem of single point failure [30]. Fig 2 offers an example of the block structure. Each block composed of two parts: block header and block body. The block header typically consists of *Version*, *PrevBlockHash*, *Timestamp*, and *MerkleRoot* [31]. The block body is responsible for storing the transaction data packaged into the block. Each block refers to the cryptographic hash of the previous block in the blockchain. If an adversary maliciously modifies the data in the blockchain, the hash pointer in the subsequent block will be incorrect [32]. This mechanism makes it possible to easily detect and reject altered blocks.

## 3. The proposed blockchain system

### 3.1 System architecture

**Fig 3:**
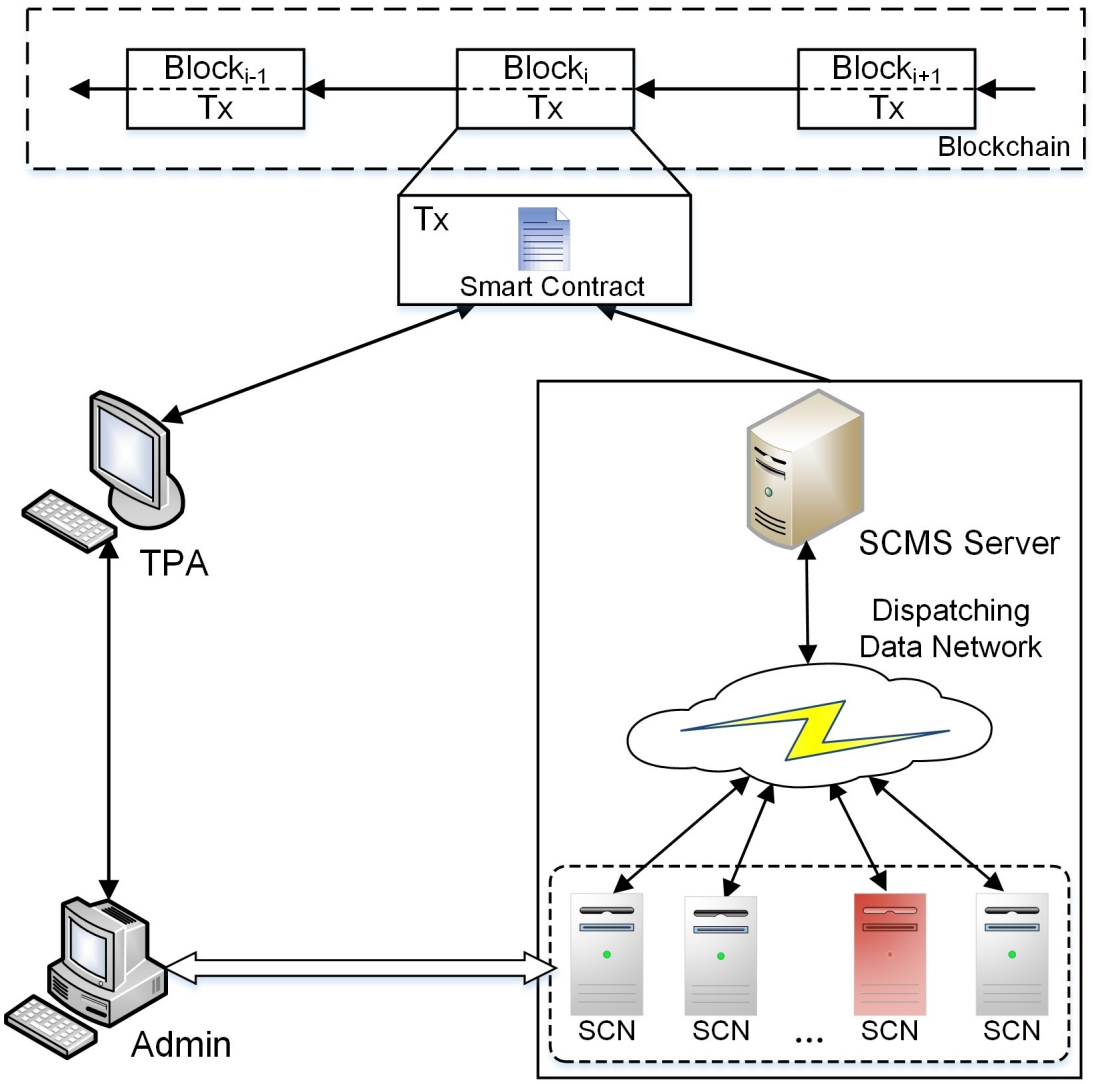
The system architecture for auditing communications of WAPS.

As shown in Fig 3, the proposed blockchain system architecture for auditing communications of WAPS contains five different entities: Administrator (Admin), Third Party Auditor (TPA), Stability Control Node (SCN), Master Node (MN) and Arbitration Node (AN).

Admin is responsible for maintaining the node list in the system, reviewing data, sending audit requests and receiving audit reports. Admin can interact with SCNs at each power plant and substation through interactive interfaces to access communication recording and auditing system of WAPS.

TPA provides audit services for users. When the Admin wants to make an audit query on communication messages, it will send an audit data query request to the TPA. TPA first reads the relevant index information of the data according to the request, and reads the corresponding data slice from the blockchain according to the index information. TPA verifies the integrity of the data by checking the hash value of the data, and finally sends the audit results to the Admin. Blockchain system can be used not only to ensure the credibility of audit results, but also to help monitor the TPA.

SCNs are the devices corresponding to each PC, IPU and IED in WAPS. The communication messages between SCNs consist of measurement data and control commands. The measurement data includes sampling values of voltage, fault information, etc. The control commands are action commands sent from the superior substation to the subordinate substation, including low frequency load shedding, tap changer control, etc. The focus of this paper is on recording and auditing control commands.

MN is a key node of the communication network of WAPS, which is located in PC. The communication messages of each SCN will be uploaded to MN. During the audit process, TPA can make an audit query to MN and get the corresponding query results to return the audit results to relevant Admin.

AN is shown as the red-colored SCN of Fig 3. It is randomly selected from SCNs in other blockchain channels. Based on fair and independent audit principles, SCNs in the same blockchain channel must contain nodes from different organizations. The AN and the nodes of the corresponding blockchain channel jointly record communication messages to prevent collective fraud of blockchain nodes.

### 3.2 Multi-chain structure and classification node mechanism

#### 3.2.1 Multi-chain structure

**Fig 4:**
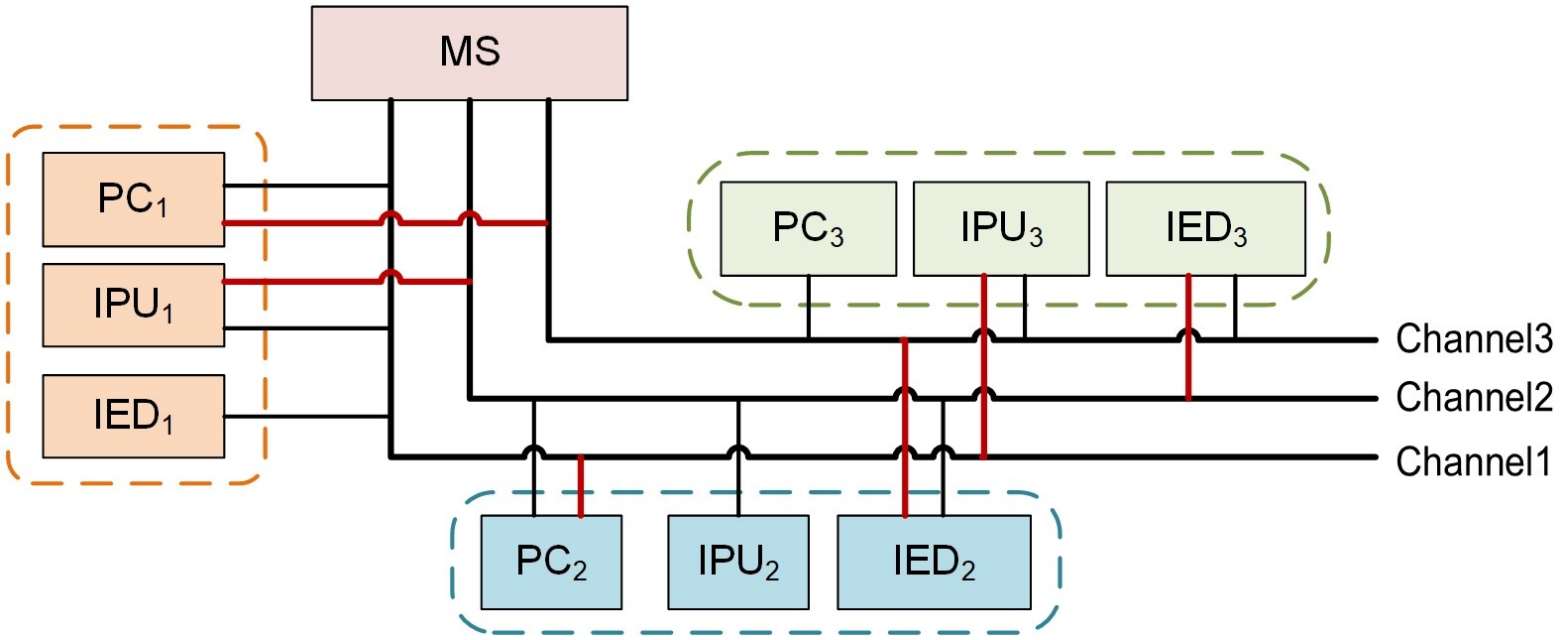
An example of multi-chain structure for WAPS.

The single-chain structure used by existing blockchains cannot meet the communication auditing requirements of multiple WAPS. In order to minimize the number of nodes on a blockchain and the amount of data stored in each node, we design a multi-chain structure considering the communication features of WAPS. Each blockchain corresponds to a communication channel composed of PC, IPU and IED, with transaction isolation and ledger isolation between chains. However, there are only three nodes in a communication channel composed of PC, IPU and IED, it is difficult to ensure the security of communication message due to the small number of nodes. Therefore, it is necessary to add two ANs to each blockchain, and ANs are randomly selected from other blockchain nodes. Since all IEDs communicate with the MN through the power dispatching data network, it is essential to add MN as a node to each blockchain. The multi-chain structure reduces the data processing pressure and resource consumption of nodes. Any business conducted on the blockchain will not be interfered by other businesses, which effectively realizes resource isolation. Fig 4 is an example of the multi-chain structure designed for auditing communication of WAPS. There are three blockchains in Fig 4. Each blockchain has six nodes, which are three SCNs (PC, IPU and IED), one MN and two ANs (the nodes connected by the red line in Fig 4 are randomly selected ANs).

#### 3.2.2 Classification node mechanism

In the current blockchain system, the types of blockchain nodes are homogeneous, and each node stores all the data of the whole network. Ever-expanding blockchain ledger requires huge storage space, which is very expensive for SCNs with limited storage resources. To mitigate this problem, we design a classification node mechanism, which classifies all nodes of the blockchain network into three types: Full Data Node (FDN), Partial Data Node (PDN) and Light Data Node (LDN).

The SCN is a LDN which stores the communication messages of the blockchain where it is located; The MN is a FDN which stores all the communication messages of the blockchain; The AN is a PDN which stores the communication messages of both the arbitrated blockchain and the blockchain where it is located. The adoption of the classification node mechanism in communication auditing of WAPS can not only save storage resources of SCNs, but also ensure the security of audit data.

### 3.3 New block structure

The data structure of the blockchain cannot adopt the data structure of the WAPS communication protocol. Considering the need of auditing, the requirement analysis of the data to be recorded on the blockchain is as follows:

1. The cause of the WAPS malfunction is usually due to the control command not being sent or received. Therefore, we need to record the function code of the control command instead of recording the complete communication message.
2. In the communication model of PC, IPU and IED, the topological relationship between substations is configured, and the adjacent hierarchical substations communicate with each other through a defined channel, so the channel numbers need to be recorded.
3. If the substation needs to communicate over the hierarchy, the communication message needs to be forwarded through the IPU at the intermediate level, and the forwarding number needs to be recorded.
4. In order to make the audit process easier to perform, it is necessary to record the time of sending the communication message and the IP of the SCN that sent the communication message.
5. In order reduce the storage burden caused by the ever-expanding blockchain ledger, we extend the block structure by adding a *MerkleRootCopy* field in the block header to store a copy of the *MerkleRoot* of the original block data. In this way, double hash chains are kept between adjacent blocks. When deleting the block body data, a valid hash chain can still be retained. The detailed scheme is presented in Section 4.

The block structure of blockchain consists of two parts: block header and block body. The data structure of block header we designed is shown in Table 1. The information in the block header is similar to other blockchain systems. The main difference is the *MerkleRoot*, where *MerkleRoot* is generated by the communication message and has a copy. Each transaction on the blockchain corresponds to a communication message that needs to be recorded. The hashing starts at the lowest level (leaf-level) nodes and is arranged and paired in order. Similarly, hashing continues at level one, which leads to hashes of hashes reaching to higher levels, until it reaches the single top to get the *MerkleRoot* of communication messages.

**Table 1:** The data structure of block header.

Value	Description
Version	Block version number
PrevBlockHash	Hash of the previous block
Timestamp	Timestamp of the block
MerkleRoot	Root of the merkle tree of communication message
MerkleRootCopy	The copy of the MerkleRoot

The data structure of block body we designed is shown in Table 2.

**Table 2:** The data structure of block body.

Value	Description
ID	Number of communication message in the block
Time	Time of sending the communication message
ChannelID	Channel number of sending the communication message
IP	Device IP that sends the communication message
FunctionCode	Function code of communication message
Trans	Forwarding number of communication message
Move	Action command in communication message

**Fig 5:**
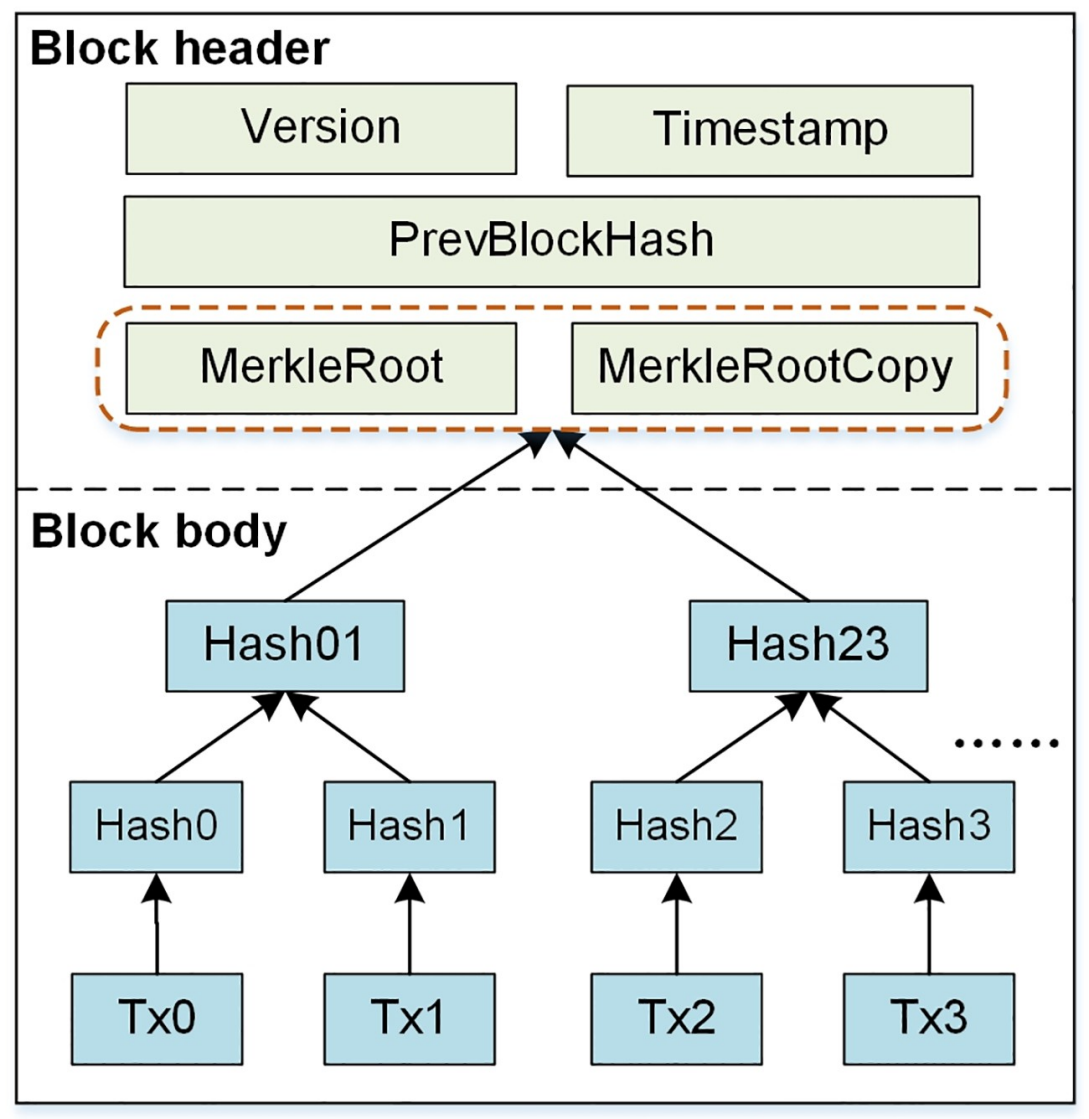
New block structure.

In summary, the new block structure that conforms to the communication protocol of WAPS is shown in Fig 5.

### 3.4 Workflow of proposed blockchain system

The workflow of the proposed blockchain system for auditing communications of WAPS includes three phases: system initialization phase, communication message on-chain phase and audit query phase.

#### 3.4.1 System initialization phase

The system initialization consists of two processes: chain structure initialization and on-chain member configuration.

1. The initialization of the chain structure first needs to configure the initial parameters of each blockchain, including the *ChannelID*, *Initime*, and *Org* of the blockchain. After completing the configuration of initial parameters, each blockchain generates the genesis block and completes the initial node to join the blockchain.
2. In order to enhance the fairness and independence of audit arbitration through multi-chain structure, the nodes in each blockchain need to be properly pre-configured by the Admin. The blockchain in which each node is located and its identity on the chain (SCN or AN) are preset into the configuration file. All nodes are instructed to join the relevant blockchain to complete the corresponding task when the system startup.

#### 3.4.2 Communication message on-chain phase

After all nodes have joined the blockchain network, the system will check the status of the ledger to synchronize the ledger in the channel, so as to perform consistent communication message on-chain with other nodes.

The SCN first broadcasts the communication message (*Info*) to the blockchain system. Then other nodes verify the validity of the audit data signature and audit data content. The MN packages the transaction data into block and broadcasts it to all nodes in the channel. All nodes on the blockchain form a consensus through the Practical Byzantine Fault Tolerance (PBFT) algorithm [33], and the communication message will be synchronized to each node. This process ensures the authenticity and tamper-proof of the data on-chain. The algorithm of the communication message on-chain is shown in Algorithm 1.

**Algorithm 1** Communication message on-chain

**Input**: communication message Info, authentication credentials C

**Output**: result of this operation Flag

1: **function** Upload(Info, C)

2: initialize the info to be sent for authentication using Info

3:  T_X_ ← RemoteAuth(info, C)

4:  **if**! Varification(T_X_) then

5:   **return** false

6:  **end if**

7:  start PBFT consensus for the infomation with T_X_

8:  **if** ! GetState(T_X_) then

9:   **return** false

10:  **end if**

11:  **return** true

12: **end function**

#### 3.4.3 Audit query phase

The audit query of communication message includes two parts: request authorization and query information. The query mode includes IP query through SCNs and time query through communication message.

When the TPA receives the audit query request, it will first read the relevant index information of the data according to the request, and read the corresponding data slice according to the index information. The TPA verifies the integrity of the data by checking the hash value of the data, and finally sends the audit results to the Admin. The algorithm of audit query is shown in Algorithm 2.

**Algorithm 2** Audit query

**Input**: query request Req, authentication credentials C

**Output**: Result

1: **function** ExcuteQuery (Req, C)

2:  **if** the audit C has no capability for the request type Req.type **then**

3:   **return** null

4:  **end if**

5:  Iterator ← GetResult(Req.state)

6:  **while** Iterator.hasNext() do

7:  Query the next related content by the index Iterator.next()

8:  Append to the array to be returned Result, Append(result, info)

9:  **end while**

10:  **return** Result

11: **end function**

## 4. Deletable blockchain

### 4.1 Motivation

The key property of the blockchain is the append-only, tampered-proof and immutable data structure. Each block contains the hash value of the previous block in its block header, thus forming an immutable hash chain [34]. The cascade effect of hash chain ensures that if an adversary modifies data in the blockchain, the hash pointer in the subsequent block will be incorrect, whereas any recalculation of the hashes in the subsequent blocks would be costly. The merits of the immutability property are obvious: once stored in the blockchain, the integrity and auditability of the data remain protected as long as the blockchain exists [35]. However, for every plus there is a minus. SCNs have limited resources in terms of storage and computation capability. Ever-expanding blockchain ledgers lead to the increasing overhead in validating and storing transaction data on the chain, causing waste of the storage resources [36]. After a safe amount of time has elapsed, it is sufficient to meet the requirements of the communication audit of wide area protection system by storing the recent audit data and deleting expired audit data. The immutability of blockchain makes it impossible to delete this expired data. Additionally, the use of blockchain applications raises various legal issues. The immutability of blockchain is in contradiction with the right to be forgotten and the right to data portability (so-called “victim rights”) defined in the new European General Data Protection Regulation (GDPR) [37]. According to this regulation, individuals have the right to delete their personal data under certain conditions. The inability to modify data stored in the blockchain prevents the development of blockchain technology. Therefore, it can be seen that the deletable blockchain has clear and urgent practical needs.

To overcome these limitations, some scholars have conducted the frontier research of revisable blockchain methods and techniques. Giuseppe Ateniese et al. [38] first proposed a rewritable blockchain scheme based on chameleon hash function in 2017. The chameleon hash function is a one-way hash function with trapdoor proposed by Krawczyk and Rabin. If you have the trapdoor information of chameleon hash function, you can easily calculate the hash collision of the input data, so that you can arbitrarily change the input of the hash function without changing the output of the hash function. Therefore, the study replaced the blockchain hash function (e.g., SHA256 in the Bitcoin system) with the chameleon hash function. If some entities obtain the secret trapdoor, they can modify the existing block without breaking the integrity of the hash chain. However, the trapdoor key was centrally managed, the user in charge the trapdoor key can modify the block data at will without any supervision, thus threatening the block data security.

Derler et al. [39] extended the method to make the modification to be performed in the transaction level rather than the block level. However, the above schemes are based on the chameleon hash function to modify the block data, and most of the existing blockchain system is based on the traditional anti-collision hash function such as SHA256, so the application scope of the above schemes is limited.

Yanli Ren et al. [40] proposed a revisable SpaceMint blockchain scheme based on POSpace consensus algorithm. The study used a secure threshold ring signature to delete all transaction data in a single block when nodes exceeding the threshold agree. However, the SpaceMint blockchain architecture is not applicable to current mainstream blockchain architecture. Moreover, the POSpace consensus algorithm requires high storage space and is not suitable for stability control devices with limited storage resources.

Alexander Marsalek et al. [41] proposed a correctable blockchain scheme that supports correction of any block data. The scheme designed a second blockchain called Correction Chain which is linked to the original blockchain called Standard Chain. The Correction Chain includes a linked list of correction blocks and is responsible for confirming the legality of the correction applied to the Standard Chain. The scheme adopted a consensus-forced vote mechanism to decentralize decisions on required data corrections. This solution is only applicable to a small number of block replacements and corrections, and it is expensive for the verifier to maintain and verify two blockchains at the same time.

Dominic Deuber et al. [42] proposed a redactable blockchain protocol for permissionless blockchain system. The protocol leveraged a consensus-based voting and is parameterised by a policy that dictates the requirements and constraints for the redactions. The protocol modified the block structure to accommodate another copy of transaction’s Merkle root, so that two hash chains are kept between adjacent blocks. The block data modification operation only destroys one hash chain, while the other hash chain still holds. However, the protocol is based on the Proof of Work (PoW) consensus mechanism [43]. PoW consensus algorithm suffers from low throughput, long confirmation time and high computation capability requirements for nodes.

As a result, existing block deletion schemes cannot be applied to WAPS with limited computation and storage resources. It is essential to research a new deletable blockchain method for communication auditing of WAPS while maintaining blockchain’s integrity and security.

### 4.2 Deletable blockchain for WAPS

**Fig 6:**
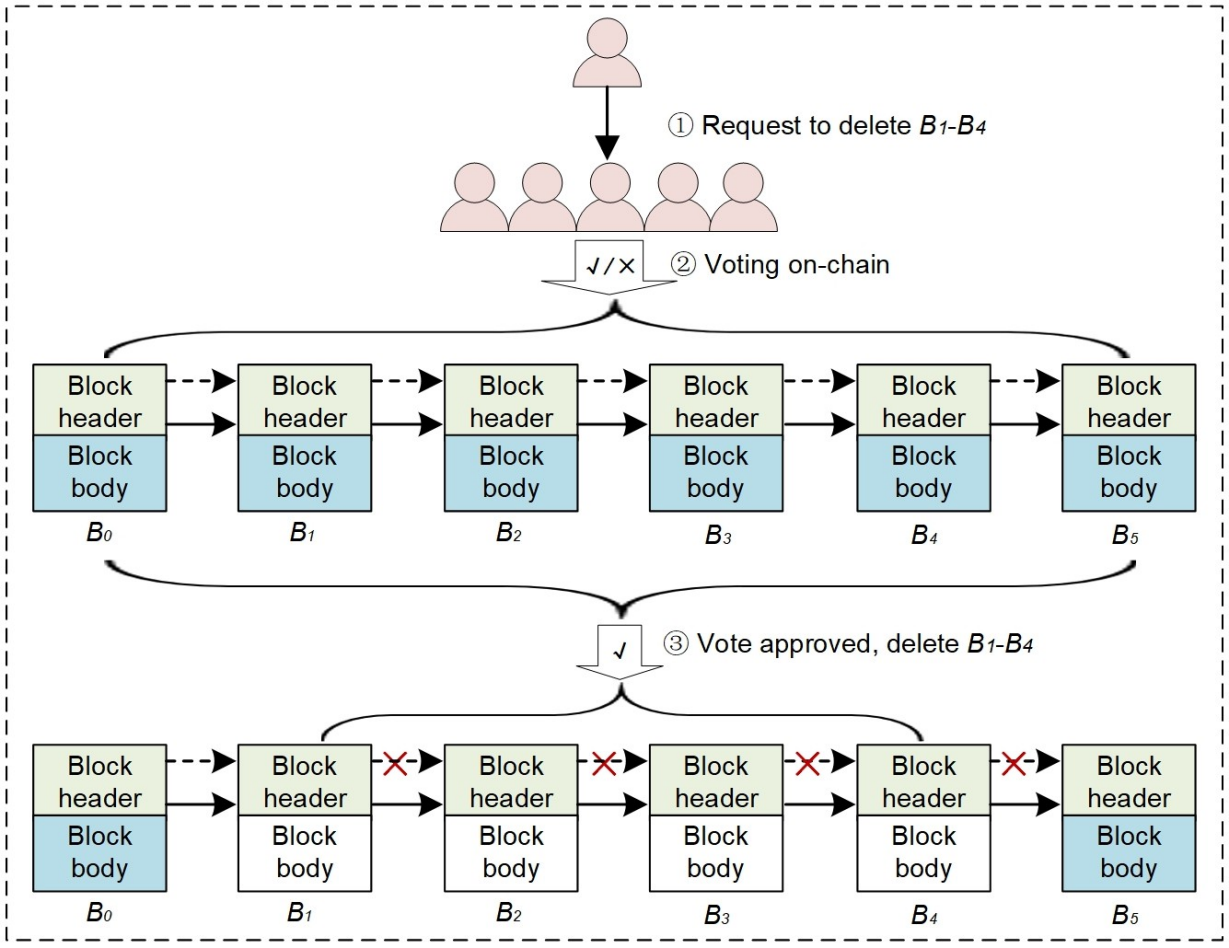
Block data deletion process based on double hash chains.

Inspired by the redactable blockchain scheme proposed by Dominic Deuber et al., we introduce it into communication auditing of WAPS to reduce the storage burden. We extend the block structure by adding a *MerkleRootCopy* field (See Fig 5 for details) in the block header to store a copy of the *MerkleRoot* of the original block data. In this way, double hash chains are kept between adjacent blocks. When deleting the block body data, a valid hash chain can still be retained. Note that the genesis block cannot be deleted and neither is the block header data, it is the block body data that is deleted each time. Instead of PoW consensus mechanism, we adopt PBFT consensus mechanism. PBFT has high performance and provides (n-1)/3 fault tolerance on the premise of ensuring liveness and safety. It is suitable for communication audit of WAPS. Fig 6 depicts the block data deletion process based on double hash chains. It includes three phases: request phase, voting phase, confirm and delete phase.

1. Request phase: The user first proposes a block data deletion request to blockchain system in the form of specific election transaction *DelT*_*x*_. *DelT*_*x*_ = {*number*, *reason*}, number is the block height of the transaction data to be deleted by the request, such as B1-B4 in Fig 6, reason is the reason for the request to be deleted.
2. Voting phase: After receiving *DelT*_*x*_, all other valid nodes are required to vote on whether or not they agree with *DelT*_*x*_, which is recorded as *ReDelT*_*x*_. *ReDelT*_*x*_ = 1, indicating that the node agrees to the deletion request; *ReDelT*_*x*_ = 0, indicating that the node does not agree to the deletion request. These votes will be written to the subsequent block of blockchain.
3. Confirm and delete phase: After the voting period Δt for the request is over, each valid node in the network can verify if the deletion request *DelT*_*x*_ is approved according to the predefined threshold (set as 2/3 in this paper). If the request is approved, the block data deletion will be executed; otherwise, the block data deletion request will be discarded.

As shown in Fig 6, after the block data of B1-B4 is deleted, the first hash chain shown in the dashed arrow is destroyed due to the change of block data, while the second hash chain shown in solid arrow formed by the *MerkleRootCopy* field is still established, thus ensuring the hash integrity of the blockchain. It can be seen that in the data consensus process, the block data will be verified according to the normal process. If the dashed hash chain of adjacent blocks is invalid, then check whether the solid hash chain formed using the *MerkleRootCopy* field is valid. If it is valid, it means that the block data has been deleted; otherwise, it means that the block has been tampered with without authorization.

### 4.3 Security analysis of deletable blockchain

The proposed deletable blockchain for auditing communications of WAPS can not only effectively delete the specified block data, but also ensure the security of blockchain.

1. Block data deletion permissions and content deletion must be jointly decided by a consensus-based voting mechanism among all verifiers. Only with the consent of the majority of nodes can the specified block data be deleted. Block data deletion does not depend on any party, thus avoiding illegal deletion by malicious users. The predefined threshold for block data deletion is 2/3. On the one hand, the higher the threshold, the more it can represent the opinions of nodes in the system, making the deletion operation safer. On the other hand, if the predefined threshold is too high, it will take more time to delete the block and affect the efficiency of the deletion operation. Therefore, we set the predefined threshold as 2/3, which can not only guarantee the security of the blockchain, but also ensure the efficiency of deletion operation.
2. The user can only submit a block data deletion request to the blockchain system in the form of a specific election transaction like *DelT*_*x*_ = {*number*, *reason*}. Except for deleting block data operation, the user cannot make any other changes to the block data, such as modifying data, inserting data, hiding data, etc.
3. The deletion of block data does not break the structure of existing blockchain. The genesis block cannot be deleted, which ensures that the source of the block data is trusted. The block deletion operation only removes the block body data from the original block, and the block header data cannot be deleted. All blocks can still be hash-linked through the block header data, ensuring that the existing blockchain structure remains invariant.
4. The deletion of block data does not break the integrity of the hash chain between adjacent blocks. We extend the block structure by adding a *MerkleRootCopy* field in the block header to store a copy of the *MerkleRoot* of the original block data. There is a double hash chain between previous and subsequent blocks. When deleting the block body data, the first hash chain is destroyed, while the second hash chain still stands. Thus, adjacent blocks still maintain a valid hash chain.

## 5. Security analysis

Blockchain has technical features of decentralization, transparency, immutability, incontestability and traceability. By using the blockchain network as a self-recording and auditable channel, blockchain data is broadcast in real time and nodes are kept synchronized. Based on this, the proposed semi-centralized blockchain system for auditing communications of WAPS has strong robustness and security.

### (1) Tamper-proof of communication message

In the proposed scheme, once the communication data is authenticated by SCNs and ANs, it will be added to the corresponding communication channel to generate a new block. Each block header contains a *PrevBlockHash* and *Timestamp* field, which forming an immutable hash chain. Any node wants to modify the block data, the hash chain in the subsequent block will be incorrect. The proposed deletable blockchain scheme requires the consent of more than 2/3 of the nodes in the whole network to delete the specified block body data. No single party can control the blockchain and no single party can modify it or shut it down. This is sufficient to meet the security requirement of blockchain.

### (2) Non-repudiation of communication message

Once the communication message of SCNs is confirmed by the consensus voting, it will be synchronized to the blockchain of each node and cannot be revoked. Each node in the communication channel stores all the data. If a node breaks down, the other nodes are still running normally, which effectively prevents the loss of audit data. Thus, blockchain can be used as a self-recording channel to realize the non-repudiation of WAPS communication message.

### (3) Resistance to Denial of Service (DoS) attack

Since all nodes of the blockchain are decentralized, each node has a complete blockchain ledger and can verify the validity of data from other nodes, so it is very difficult for DoS attack against the blockchain. Even if an attacker maliciously breaches one node, the remaining nodes can maintain the entire blockchain system normally and can effectively resist DoS attack.

### (4) Resistance to single point of failure

The blockchain is considered reliable due to the ledger of blockchain needs to be maintained by all blockchain nodes. Even if one node goes offline, the ledger is still readily available to all other valid nodes in the network, so the blockchain lacks a single point of failure.

## 6. Implementation and performance evaluation

To evaluate the effectiveness and performance of the proposed scheme, we used Hyperledger Fabric v1.4.0 to implement an experimental prototype system. Hyperledger Fabric is primarily used to build the blockchain environment needed for the experiment, including node configuration, channel creation, smart contract deployment and operation. We implement the smart contract based on the Go language to process the business logic approved by the nodes on the blockchain.

The experiments are conducted on Ubuntu 18.04.3 LTS x86 system, with Intel(R) Core(TM) i5-6300HQ CPU @ 2.5 GHz processor and 12 GB memory. The experimental prototype system consists of one Admin, one TPA, one MN server and several SCNs. All of them are added in the same channel in Hyperledger Fabric. There is a chaincode made up of two functions called Query and Invoke. The Query function is used to obtain data from the task publisher. The Invoke function is used to provide feedback on the next task. The Admin uses one Invoke function to publish task and one Query function to obtain the audit results. The TPA uses one Query function to obtain information, one Invoke function to own the task and one Invoke function to respond to the task. The MN server uses one Query function to confirm whether a user has asked the TPA to issue an audit request.

Since there is no known scheme that applies existing blockchain to the communication recording and auditing of WAPS, the proposed scheme lacks a blockchain audit scheme that can be directly compared. The metrics that affect the time latency in the auditing process include the number of peers and the size of block. Only one parameter is changed at a time, so any change in performance will be based entirely on that parameter. To this end, we highlight the strengths of the proposed scheme in steps through the following experiments.

**Fig 7:**
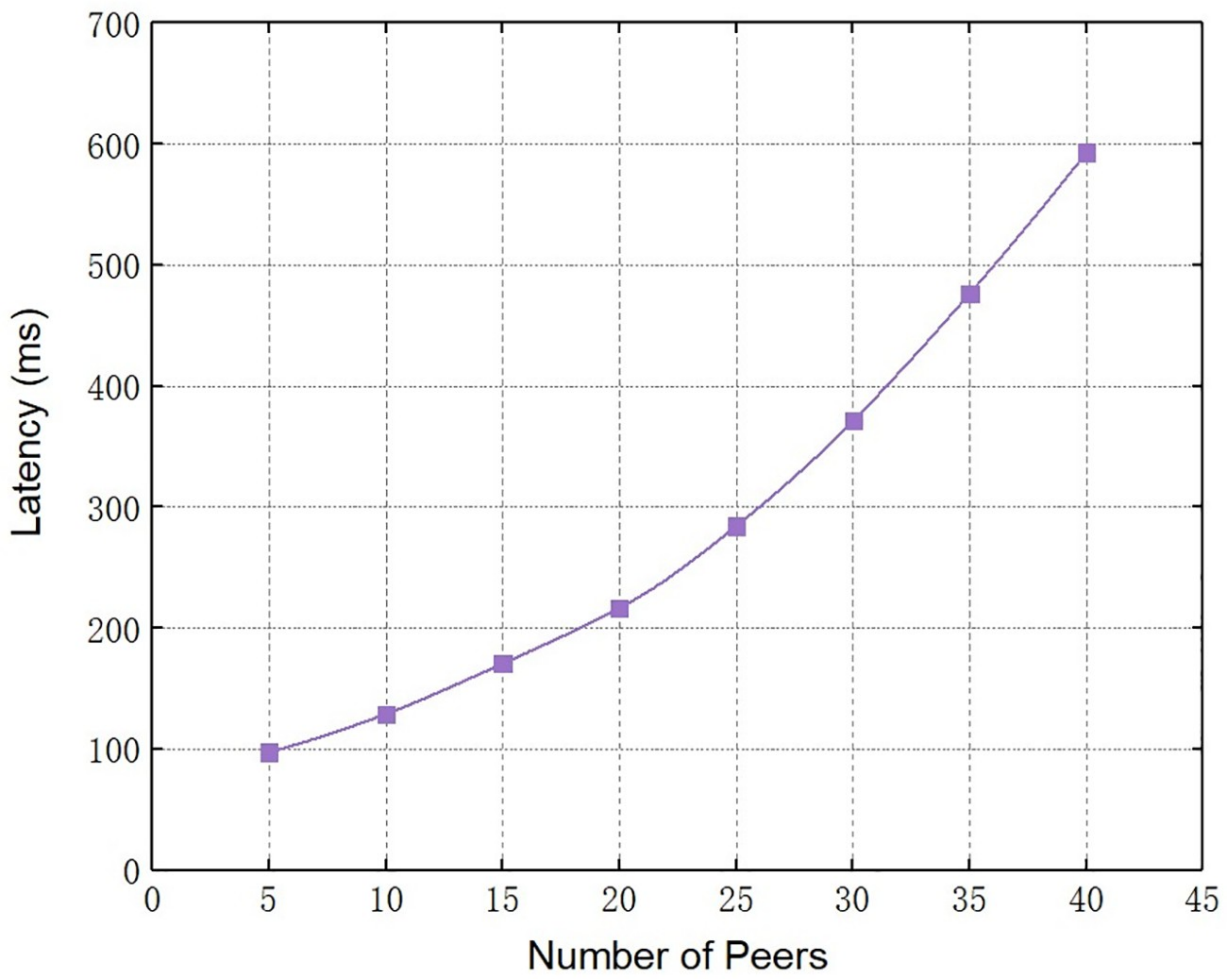
The effect of number of peers on audit query latency.

First, we test the time latency of the audit query on a single blockchain. We set the size of the block to 256 bytes, and gradually increased the number of peers from 5 to 40, thus getting the effect of the number of peers on the audit query latency of the system. The specific experimental result is shown in Fig 7. It can be seen that as long as the number of peers remains within 20, the time latency of audit query will not change drastically. As the number of peers exceeds 20, the time latency of audit query increases considerably. Due to the multi-chain structure we designed distributes the nodes of the blockchain system across multiple blockchains, so that the number of nodes on each blockchain is no more than 20. The time latency of audit query in our scheme is lower than in schemes such as [22] that adopt the single-chain structure. This experimental result highlights the advantages of the multi-chain structure designed in Section 3.2.1.

**Fig 8:**
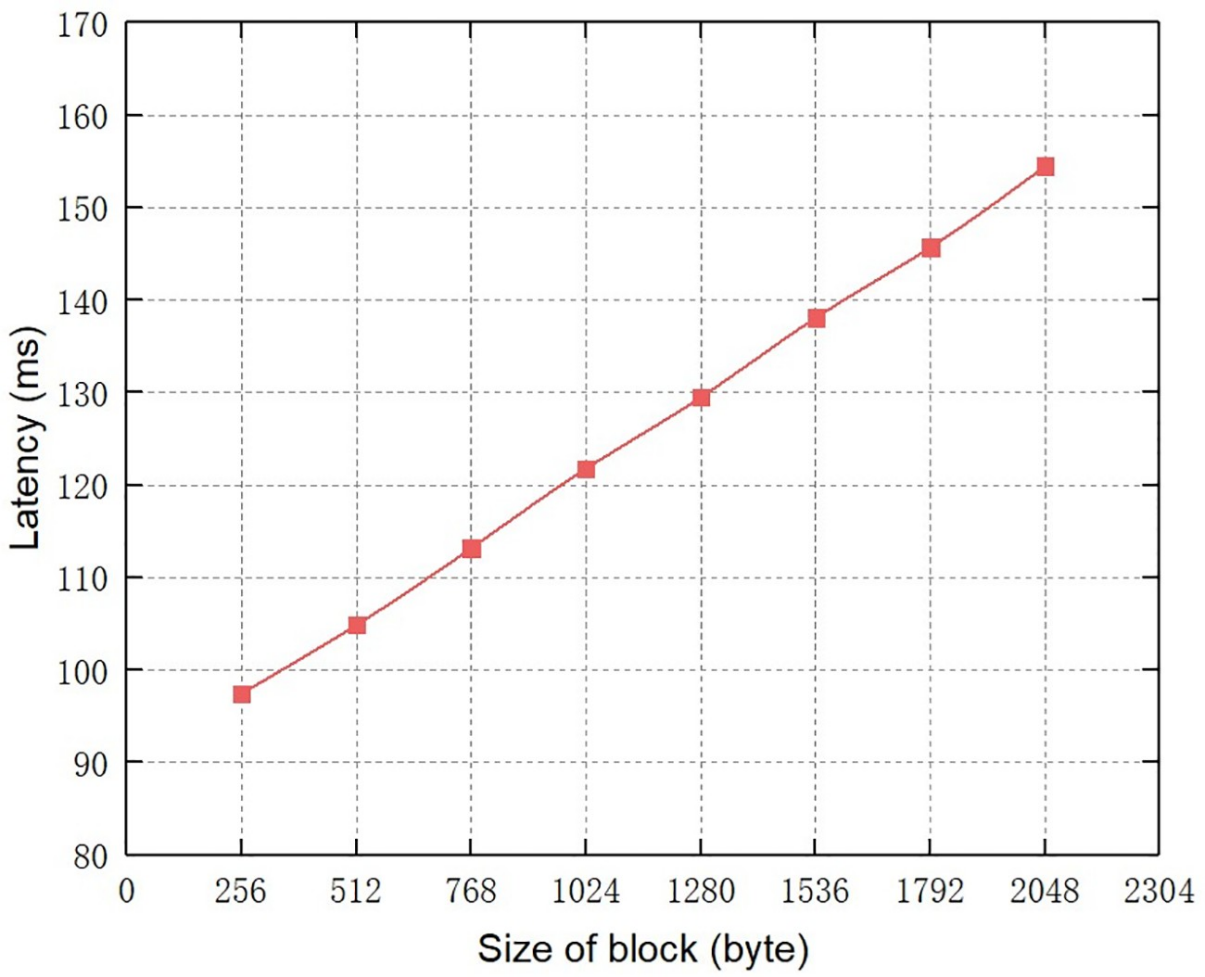
The effect of size of block on audit query latency.

Then, we set the number of peers to 5, and gradually increase the size of block from 256 bytes to 2048 bytes, thus getting the effect of the size of block on the audit query latency of the system. The specific experimental result is shown in Fig 8. It can be seen that as the size of block increases, the time latency of audit query almost grows linearly. Due to the classification node mechanism we designed can significantly reduce the size of blockchain ledger stored by nodes except MN, only the function codes of control commands rather than all communications are recorded on the blockchain, so the proposed blockchain system allows for smaller size of block, thus reducing the time latency of audit query. The time latency of audit query in our scheme is also lower than that in [18]. This experimental result highlights the advantages of the classification node mechanism designed in Section 3.2.2 and the new block structure in Section 3.3.

Since the blockchain network is an ever-expanding system in terms of size, we propose a deletable blockchain scheme to delete expired audit data. It is clearly apparent that the operation of deleting block data will bring extra overhead. To demonstrate the viability of our approach, we tested the time overhead introduced by the deletable blockchain compared to the standard blockchain. We generated random deletable blockchains containing 100 to 1000 blocks. Each block contains transaction data consisting of 100 bytes that are deleted during the experiment. We set the ratio of deleting transaction data to one-tenth of all transactions. We assumed that this is a reasonable and realistic number in our experiments. Then, we measured the time overhead introduced by the deletable blockchain of different blockchain lengths compared with the standard blockchain.

**Fig 9:**
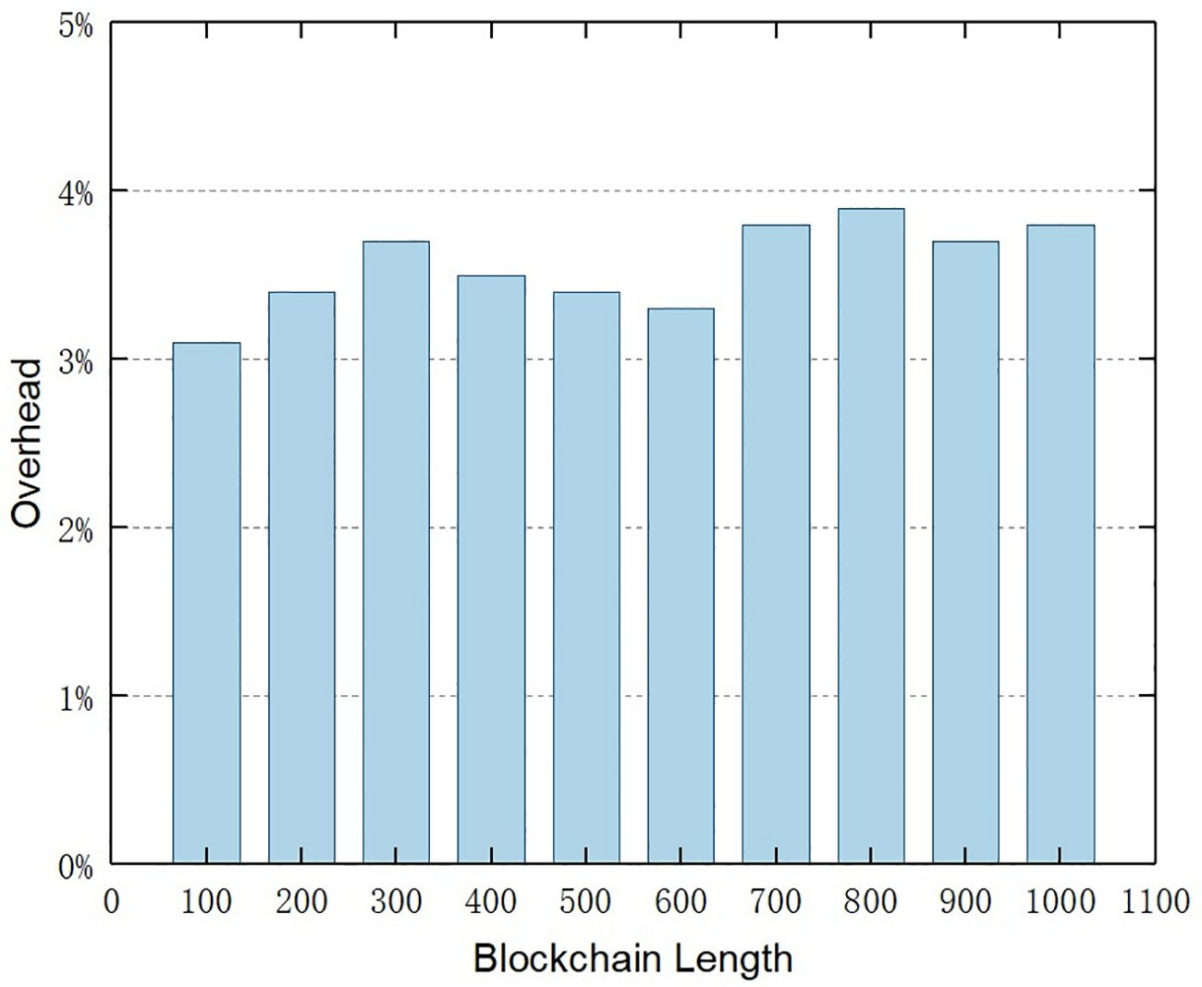
Time overhead introduced by the deletable blockchain of different blockchain lengths compared with the standard blockchain.

Fig 9 illustrates the overhead imposed by the deletable blockchain for different blockchain lengths. Compared with the standard blockchain, the overhead imposed by our proposed deletable blockchain is less than 4 percent, which has a minor impact on the overall performance. As the size of the chain grows, the overhead tends to stabilize. This result meets our expectations, since the time overhead mainly comes from maintaining and increasing the voting counts during the voting period, as well as the network speed. This experimental result highlights the advantages of the deletable blockchain designed in Section 4.

In summary, the experimental results show that the time overhead of our scheme is acceptable, and the additional overhead brought by the deletable blockchain is limited. The proposed scheme is feasible and effective in communication message recording and auditing of WAPS.

## 7. Conclusion

In this paper, we propose a semi-centralized blockchain system with multi-chain for auditing communications specifically for the system architecture and management requirements of WAPS. We utilize the blockchain network as a self-recording channel to achieve tamper-proof and non-repudiation verification interaction. Considering the communication auditing requirements of multiple WAPS, we design a multi-chain structure and classification node mechanism, which can effectively reduce computation resource consumption and save storage resources of system nodes. We redesign the block structure and transform the communication records and audit logs into a blockchain-compatible data structure for storage. To reduce the storage burden caused by the ever-expanding blockchain ledger, we propose a deletable blockchain while maintaining the integrity and security of blockchain. Security analysis and experiment results show that the proposed blockchain system is feasible and can effectively support the secure, transparent, tamper-proof and traceable communication recording and auditing of WAPS. The communication messages recorded on the blockchain can provide effective evidence for the investigation of accidents.

As for the further improvements to this work, we plan to research the lightweight cryptographic technology in the blockchain system to provide privacy protection for data on-chain. Moreover, the proposed semi-centralized blockchain system for auditing communications of WAPS can be used for attack detection. By analyzing and monitoring the communication messages recorded on the blockchain, the abnormalities and malicious attacks in the network can be quickly detected. We will further study the specific methods and security strategies of attack detection by means of semi-centralized blockchain system.

## Supporting information

S1 File (ZIP)
