# Supplementary figures and images for: A semi-centralized blockchain system with multi-chain for auditing communications of Wide Area Protection System
Source: PLoS One. 2021 Jan 22;16(1):e0245560. doi: 10.1371/journal.pone.0245560 (PMC7822322; doi:10.1371/journal.pone.0245560)

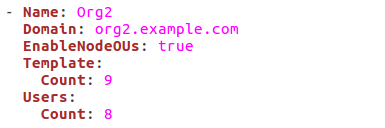

Supplement: S1 File — (ZIP) [file pone.0245560.s001.zip › Supporting Information/Org.png]

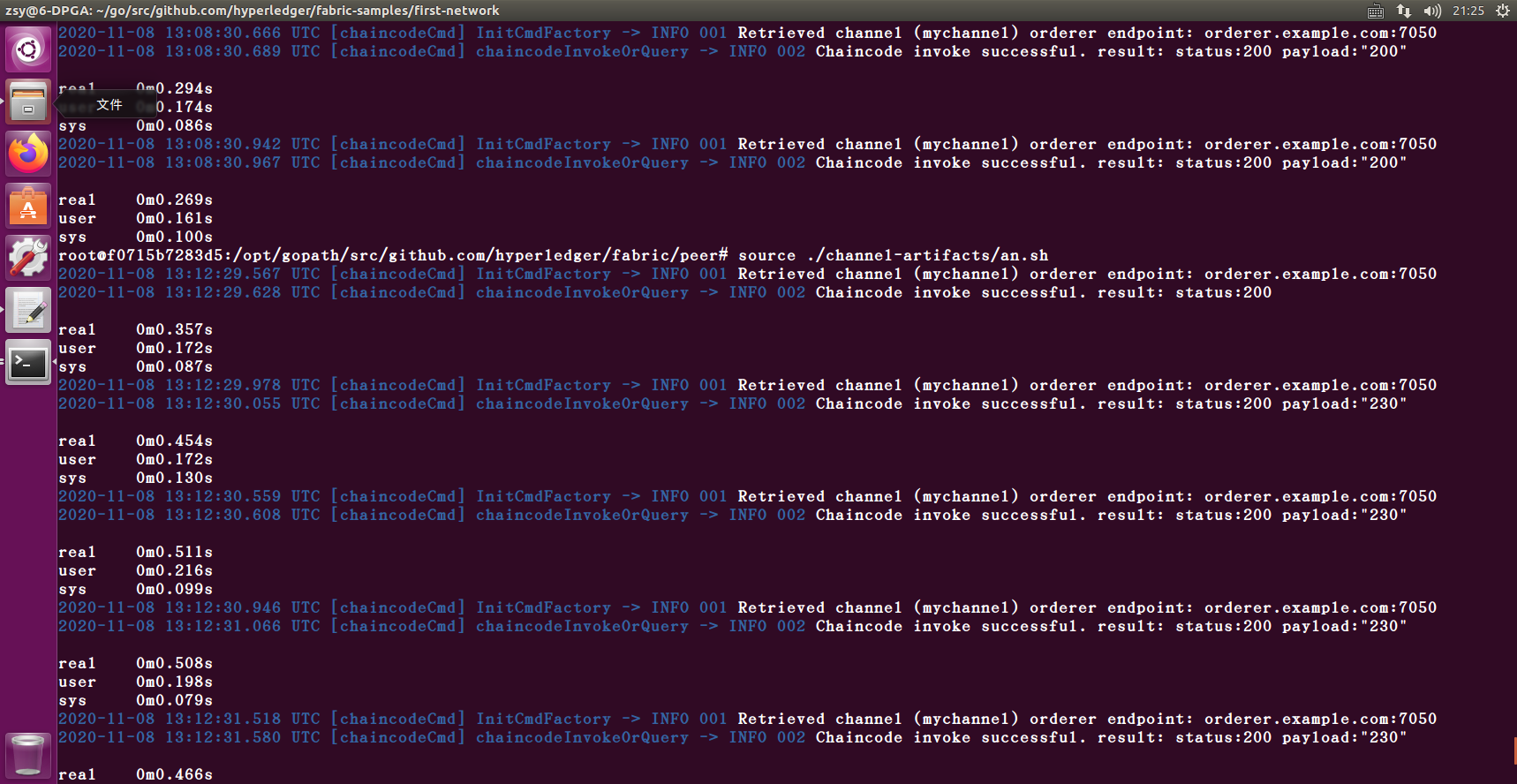

Supplement: S1 File — (ZIP) [file pone.0245560.s001.zip › Supporting Information/Query latency1.png]

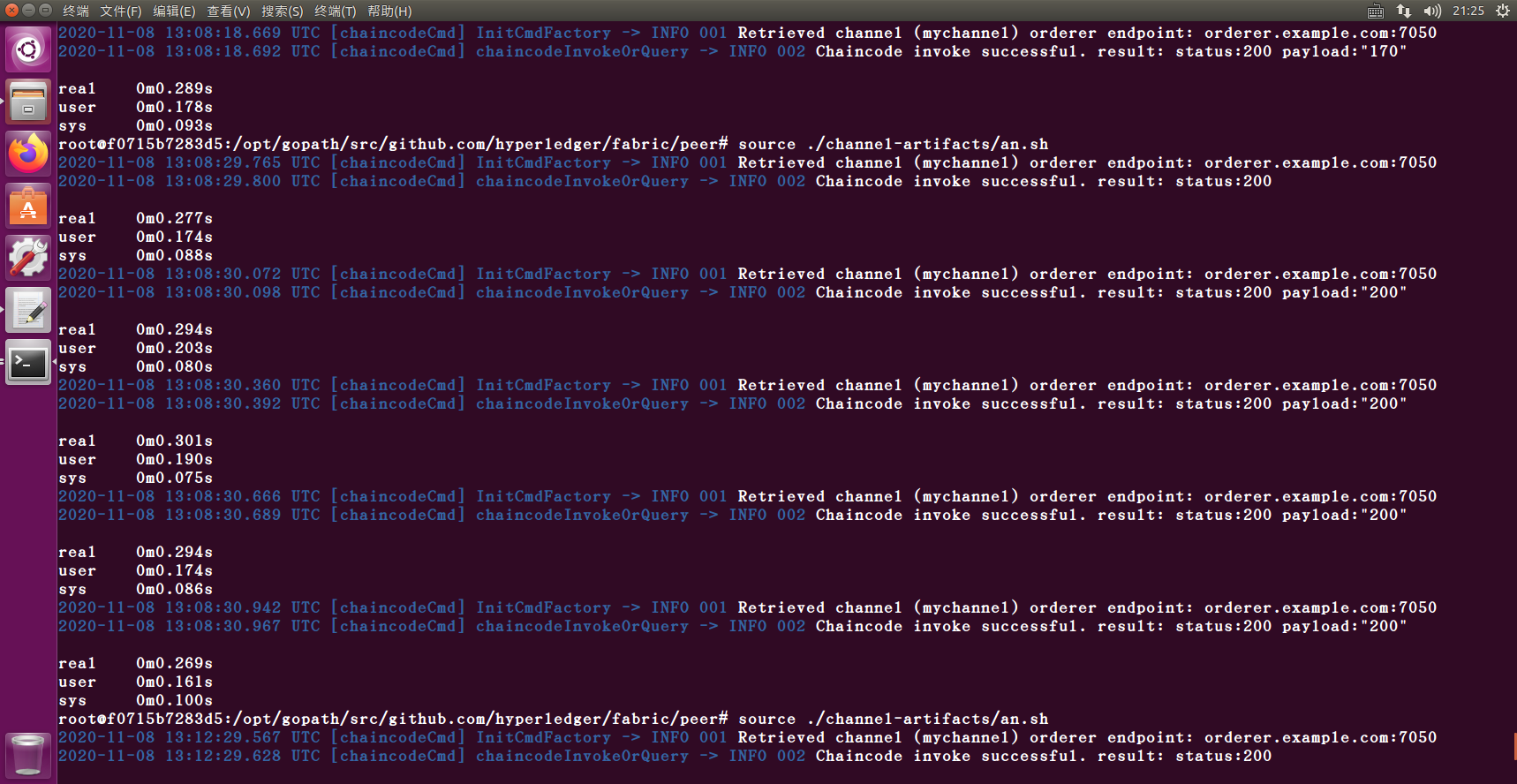

Supplement: S1 File — (ZIP) [file pone.0245560.s001.zip › Supporting Information/Query latency2.png]
